# Identification of a Novel PPAR-γ Agonist through a Scaffold Tuning Approach

**DOI:** 10.3390/ijms19103032

**Published:** 2018-10-04

**Authors:** Hyo Jin Gim, Yong-Sung Choi, Hua Li, Yoon-Jung Kim, Jae-Ha Ryu, Raok Jeon

**Affiliations:** Drug Information Research Institute, Research Institute of Pharmaceutical Sciences, Sookmyung Women’s University, Cheongpa-ro 47-gil 100, Yongsan-gu, Seoul 04310, Korea; hjgim83@gmail.com (H.J.G.); uriys2@gmail.com (Y.-S.C.); cooldog227@hotmail.com (H.L.); yoonjungkim73@gmail.com (Y.-J.K.); ryuha@sookmyung.ac.kr (J.-H.R)

**Keywords:** PPAR, agonist, indole, scaffold, tuning

## Abstract

Peroxisome proliferator-activated receptors (PPARs) are important targets in metabolic diseases including obesity, metabolic syndrome, diabetes, and non-alcoholic fatty liver disease. Recently, they have been highlighted as attractive targets for the treatment of cardiovascular diseases and chronic myeloid leukemia. The PPAR agonist structure is consists of a polar head, a hydrophobic tail, and a linker. Each part interacts with PPARs through hydrogen bonds or hydrophobic interactions to stabilize target protein conformation, thus increasing its activity. Acidic head is essential for PPAR agonist activity. The aromatic linker plays an important role in making hydrophobic interactions with PPAR as well as adjusting the head-to-tail distance and conformation of the whole molecule. By tuning the scaffold of compound, the whole molecule could fit into the ligand-binding domain to achieve proper binding mode. We modified indol-3-ylacetic acid scaffold to (indol-1-ylmethyl)benzoic acid, whereas 2,4-dichloroanilide was fixed as the hydrophobic tail. We designed, synthesized, and assayed the in vitro activity of novel indole compounds with (indol-1-ylmethyl)benzoic acid scaffold. Compound **12** was a more potent PPAR-γ agonist than pioglitazone and our previous hit compound. Molecular docking studies may suggest the binding between compound **12** and PPAR-γ, rationalizing its high activity.

## 1. Introduction

Peroxisome proliferator-activated receptors (PPARs) are transcription factors that belong to the nuclear receptor superfamily. There are three subtypes of PPARs, designated as PPAR-α, -γ, and -δ(β), which exhibit different tissue expression profiles and modulate specific physiological functions. PPARs play a critical role in the regulation of multiple genes that regulate glucose and lipid metabolism and energy homeostasis [1,2,3,4,5]. Because they are involved in multiple metabolic pathways, PPARs are important molecular targets for the development of new drugs for metabolic diseases, such as obesity, metabolic syndrome (MetS), diabetes, and non-alcoholic fatty liver disease (NAFLD) [6,7,8,9,10,11]. Recent studies have shown that activation of PPARs not only regulates metabolic pathways but also mediates various biological effects related to inflammation, apoptosis, oxidative stress, and vascular function [12,13,14,15,16]. These effects seem to be beneficial in other disease conditions. It has been reported that the anti–diabetic PPAR agonists are also effective in cardiovascular disease (CVD) [17,18,19], thyroid [20,21], colorectal [21], and lung cancer [22] and chronic myeloid leukemia (CML) [23,24,25].

**Figure 1:**
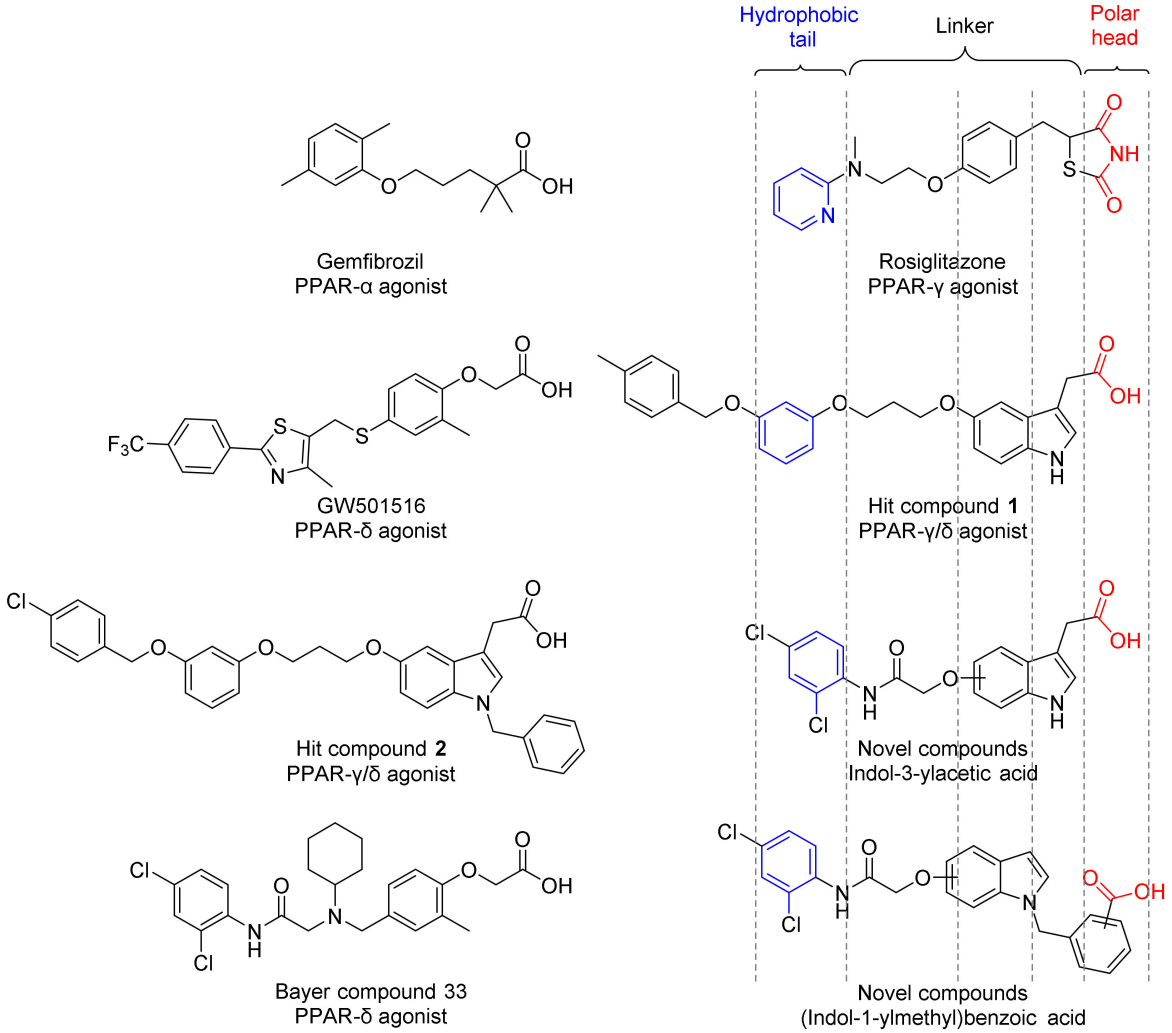
Structures of the representative PPAR agonists and newly designed compounds.

Currently, the PPAR-α agonists, fibrates (e.g., gemfibrozil, Figure 1) are used to treat dyslipidemia, whereas the PPAR-γ agonists, thiazolidinediones (TZDs; e.g., rosiglitazone, Figure 1) are used to treat type 2 diabetes mellitus (T2DM). However, the use of TZDs is associated with various adverse effects, particularly weight gain, bone fractures, cardiovascular complications, and edema [26,27,28]. No drugs in the market have been identified to target PPAR-δ(β). Continuous efforts are being made by many research groups and pharmaceutical companies worldwide to develop potent and safe therapeutic agents for the treatment of PPAR-associated diseases. In particular, they aim to develop pan, dual, or selective agonists of the three PPAR subtypes [29,30,31,32,33]. Representative PPAR agonists are depicted in Figure 1.

All subtypes of PPAR share structural and functional feature similar to those of other nuclear receptors. Crystal structures of human PPAR-α, PPAR-γ, and PPAR-δ(β) have revealed a common three-dimensional structure of the ligand binding domains (LBDs). An antiparallel sandwich of 12 α-helices (helix 1 to helix 12) and a three-stranded antiparallel β-sheet are forming a large ligand binding cavity in the core of the LBD. The central cavity spans between the AF-2 domain within C-terminal α-helix 12 and the three-stranded antiparallel β-sheet [34]. Interestingly, despite a common general structure of the LBD, ligand binding to PPARs shows both species and isotype specificities. The ligands for PPAR-α or PPAR-γ should be able to adopt a U-shaped conformation and an L-shaped conformation for PPAR-δ(β) [35].

**Figure 2:**
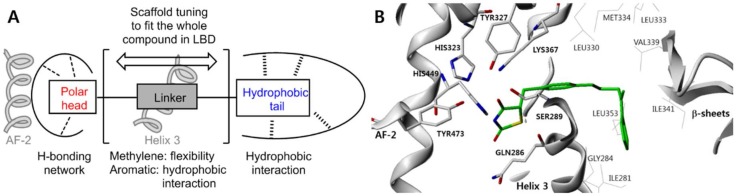
(**A**) Schematic features of PPAR agonists and the concept of the novel target compounds; (**B**) Crystal structure of PPAR-γ LDB and rosiglitazone (PDB code: 2PRG). Only the critical amino acids are displayed and labeled. Polar residues for the key interaction (capped stick) and hydrophobic residues (gray line) are depicted. Rosiglitazone is displayed in green and oxygen, nitrogen and sulfur atom is marked in red, blue and yellow respectively.

A typical PPAR agonist consists of three parts: a hydrophobic tail moiety, a polar head group (usually bearing a carboxylic acid functionality) and a linker which consists of flexible methylene units and an aromatic ring (Figure 2A). The acidic head group is crucial for PPAR activation. It forms an H-bonding network with a part of the PPAR that mainly contains the critical polar residues, such as Gln286, Ser289, His323, Tyr327, Lys367, His449, and Tyr473 in PPAR-γ (see Figure 2B). The TZD moiety (acidic head group of rosiglitazone) makes several specific interactions with amino acids. The carbonyl groups of the TZD make hydrogen bonds with His323 and His449. His323 forms a secondary hydrogen bond with Tyr473 in the AF-2 domain. The nitrogen of the TZD head group is within hydrogen-bonding distance of the hydroxyl group of Tyr473. Lys367 forms another secondary hydrogen bond with the ligand, at residue His449. The conformation of the TZD head group and the participating amino acids are fixed by these primary and secondary hydrogen bonds [34]. This H-bonding network stabilizes the conformation of the AF-2 domain allowing it to bind with the coactivator proteins. Therefore, the presence of the acidic head in the right position is important in the development of PPAR agonists. Another part of the PPAR agonists is the hydrophobic moiety that mainly interacts with the hydrophobic residues in the LBD. The hydrophobic tail occupies the large cavity of the LBD by interacting with the hydrophobic residues, such as Ile281, Gly284, Ile341, and Leu353 in PPAR-γ (see Figure 2B). The linker wraps the central helix 3 (H3) and interacts with the surrounding hydrophobic residues. In addition, it acts as a linker between the acidic head and hydrophobic tail groups to allow the fitting of the whole compound into the LBD, where proper interactions could be achieved. Therefore, fine tuning of the linker to adjust the atomic distance or three-dimensional arrangement is a powerful approach for structural optimization to increase the biological activity of compounds.

Previously, we identified a series of (alkoxyindol-3-yl)acetic acids as PPAR agonists [36,37,38]. Through stepwise structural modification and optimization, benzyloxy-containing indol-3-ylacetic acid analogs (**1** and **2**) were identified as PPAR-γ/δ dual agonists. Compound **2** lowered blood glucose, insulin, and glycated hemoglobin (HbA1c) levels without causing weight gain; additionally, it reduced the accumulation of lipids and the size of the adipocytes in db/db mice. These findings indicated the potential of the indol-3-ylacetic acid as a core scaffold for anti-diabetic and anti-dyslipidemic drugs. In our previous series of PPAR agonists, the indole group held the critical polar functional group. In addition, the *N*-benzyl group of the indole ring significantly affected the PPAR activity. Hence, we performed further modifications of the indole-based PPAR agonists.

Based on the indol-3-ylacetic acid scaffold, we introduced the dichloroanilide group into the hydrophobic tail to increase the potency and selectivity. Some reported compounds with the 2,4-dichloroanilide moiety showed a significant PPAR agonist potency, and even small changes to the 2,4-dichlorophenyl substitution resulted in a marked change in potency. For example, Bayer compound **33** (Figure 1) showed a potent and selective activity as PPAR-δ agonist with EC_50_ value of 3 nM and >1000-fold selectivity. The 2,4-disubstituted anilines were superior to other substitution patterns [39]. Luckhurst et al. drew inspiration from Bayer compound **33** and combined the anilide portion with isoindoline, tetrahydroisoquinoline, and benzazepine scaffolds. The structure-activity relationship study confirmed that the 2,4-dichloroanilide compounds were the most potent of any other derivatives [40,41]. Although interactions between 2,4-dichloroanilide and LBD of PPAR is not specified in those studies, 2,4-dichloroanilide moiety is multifunctional which can make many kinds of interactions including aromatic pi-interaction, halogen-hydrogen bonding, and hydrogen bonding through aromatic phenyl, dichloro and amide group, respectively. Furthermore, newly designed 2,4-dichloroanilide substituted analogues show proper U-shaped binding mode similar to rosiglitazone in our preliminary molecular modeling study.

For further structural optimization, including re-positioning of the critical acidic head group, we set the change in the hydrophobic tail group as a starting point for our journey (Figure 2).

## 2. Result and Discussion

**Scheme 1:**
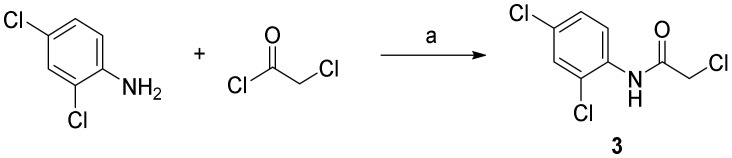
Reaction conditions: (a) triethylamine, CH_2_Cl_2_, 0 °C.

**Scheme 2:**
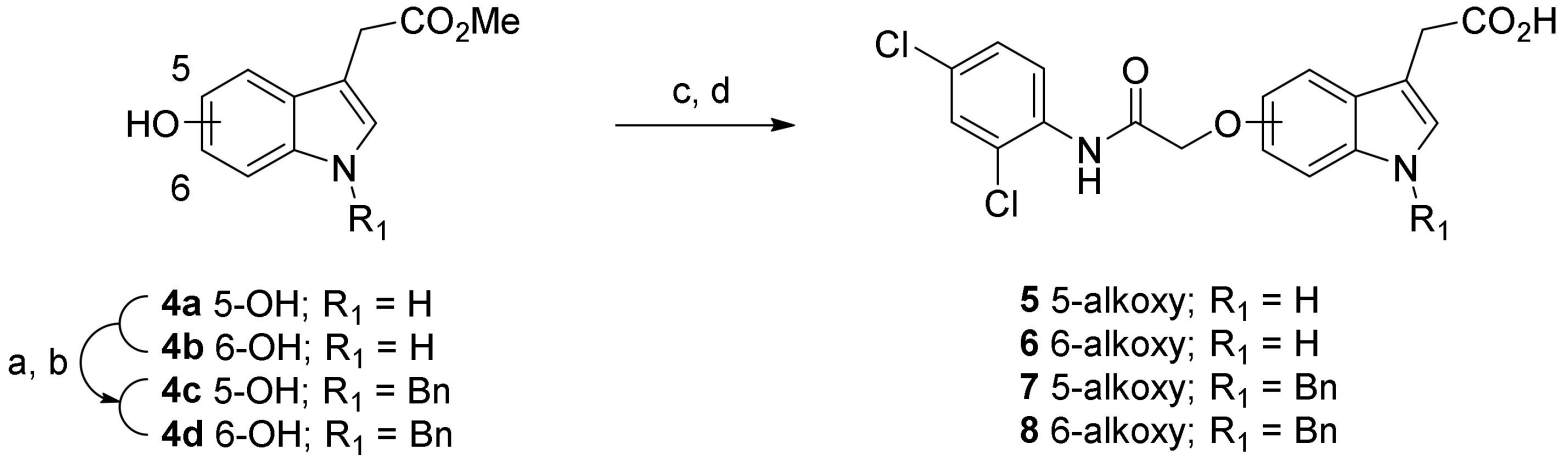
Reaction conditions: (a) benzyl bromide, Cs_2_CO_3_, DMF, 80 °C; (b) H_2_, Pd/C, EtOAc/EtOH, RT; (c) compound 3, Cs_2_CO_3_, MeCN, RT; (d) LiOH·H_2_O, THF/MeOH/H_2_O, RT.

**Scheme 3:**
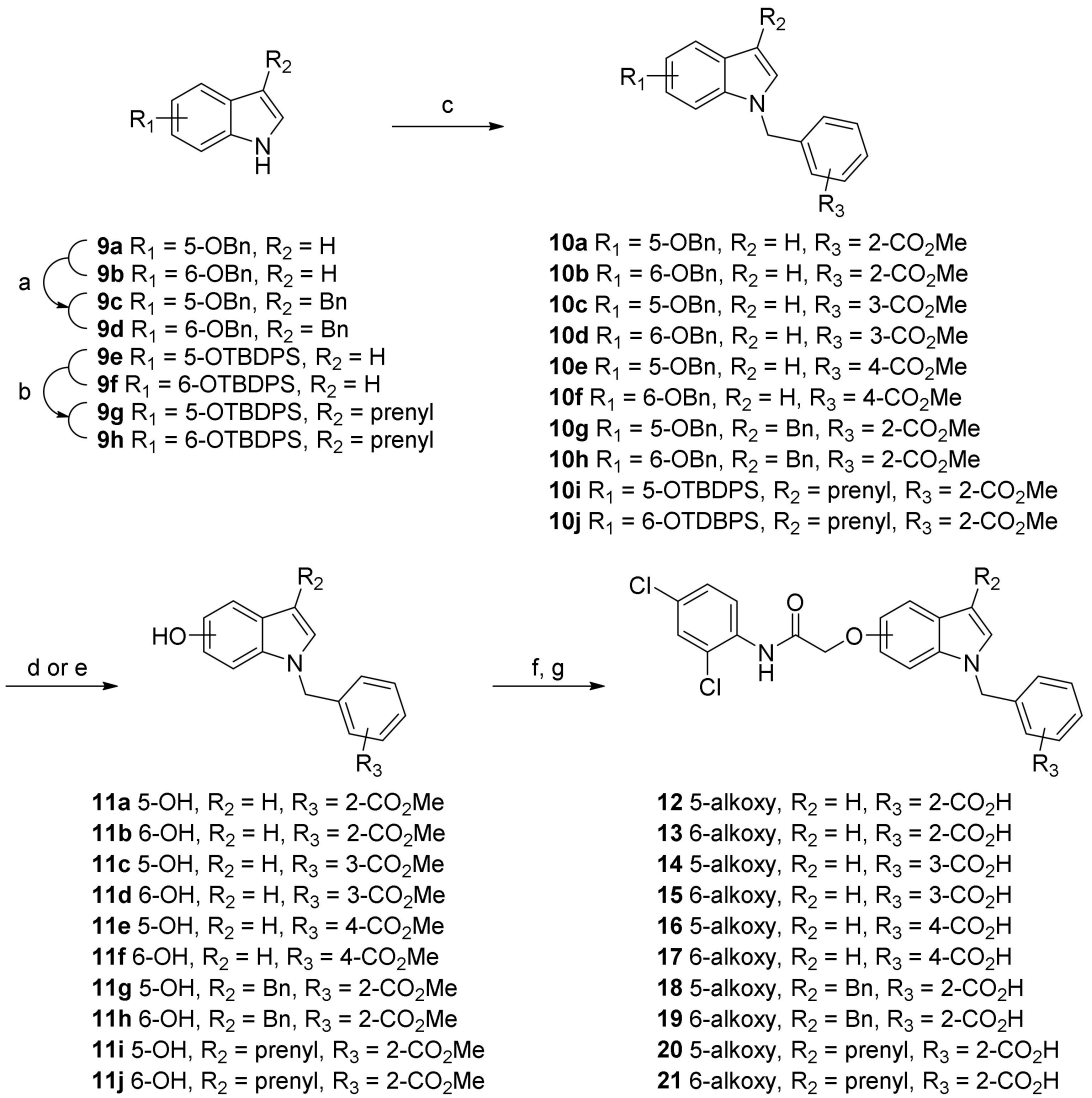
Reaction conditions: (a) benzyl alcohol, KOH, xylene, reflux; (b) prenyl bromide, Na_2_CO_3_, MeCN/H_2_O (9:1), RT; (c) methyl 2-, 3- or 4-(chloromethyl)benzoate, NaH, THF, reflux; (d) H_2_, Pd/C, EtOAc/EtOH, RT; (e) TBAF, THF, RT; (f) compound **3**, Cs_2_CO_3_, MeCN, RT; (g) LiOH·H_2_O, THF/MeOH/H_2_O, RT.

The synthesis of dichloroanilide-linked indole compounds is illustrated in Scheme 1, Scheme 2 and Scheme 3.

For the introduction of the dichloroanilide moiety as a hydrophobic tail, 2,4-dichloroaniline and 2-chloroacetyl chloride underwent a direct substitution reaction to yield 2-chloro-*N*-(2,4-dichlorophenyl)acetamide (**3**; Scheme 1).

Hydroxyindol-3-ylacetic acid methyl ester compounds (**4a**,**4b**) were prepared as previously described [32]. The amino and hydroxyl groups of the indole were benzylated under basic conditions. The *O*-benzyl group was selectively deprotected in the following hydrogenation step. Then, the developed free hydroxyl group was conjugated with compound **3**. The methyl esters were hydrolyzed using LiOH to yield the corresponding carboxylic acid compounds (**5**–**8**; Scheme 2).

The starting *O*-benzyl and *tert*-butyldiphenylsilyl (TBDPS)-protected hydroxyindoles were synthesized under typical conditions. Compounds with the 3-benzyl group were prepared by reaction of compound **9a**,**b** with benzyl alcohol, whereas the prenyl group was introduced at the 3-position of the *O*-TBDPS-protected compounds (**9e**–**f**) using prenyl bromide. Compounds with methyl carboxylate groups at various positions of the *N*-linked benzyl group were directly synthesized by substitution reaction between the amino group of the indole and the 2-, 3-, or 4-(methoxycarbonyl)methyl-substituted benzyl chloride. The *O*-benzyl and TBDPS protecting groups were removed by conventional hydrogenation and tetrabutylammonium fluoride (TBAF), respectively. *O*-Alkylation and hydrolysis of the esters yielded the target compounds (**12**–**21**;Scheme 3).

The structures of all the target compounds (**5**–**8**, **12**–**21**) were confirmed by their proton nuclear magnetic resonance (^1^H NMR) spectra and high-resolution mass spectrometry (HRMS), and they were cross-checked against the structures of their methyl ester precursors (**P5**–**8**, **P12**–**21**) which were characterized by the ^1^H and ^13^C NMR spectra. Analytical data are provided in the Materials and Methods section.

The PPAR activities of the synthesized dichloroanilide-linked indole analogs were assessed using a standard cell-based transactivation assay in CV-1 cells. GW7647 [42], pioglitazone, and GW0742 [43] were used as positive controls for PPAR-α, -γ, and -δ, respectively. These three positive controls presented the maximum activation for each PPAR subtype. The activity of the compounds was presented as the activation percentage (%) relative to the maximum values of the positive controls at the indicated concentrations. PPAR-α, -γ, and -δ transactivation activities of the tested compounds are summarized in Table 1 and Table 2.

Compounds **5** and **6** were the simplest indoly-3-ylacetic acids containing the dichloroanilide tail at the 5- and 6- positions, respectively. However, these compounds showed minimum agonistic activities to PPARs. Their *N*-benzylated analogs (**7**,**8**) showed an increase in the activity, which is in line with the trends observed in a previous study. However, their activities were much lower than those of the previous hit compounds **1** and **2** (134.4 and 100.5% activity on PPAR-γ, respectively) [33]. This decline in the activity could be explained by the length and rigidity of the linker between the hydrophobic tail and indole ring. As shown in Figure 1, the linker in compounds **1** and **2** consists of four rotatable bonds between the two planar aromatic ring systems. Rosiglitazone also has three bonds, which are freely rotatable. In contrast, compounds **5**–**8** have three rotatable bonds; however, two of them (same as three atoms) are linked to a planar carbonyl system. This non-flexibility could result in a difference in the docking poses of the compounds. 

**Figure 3:**
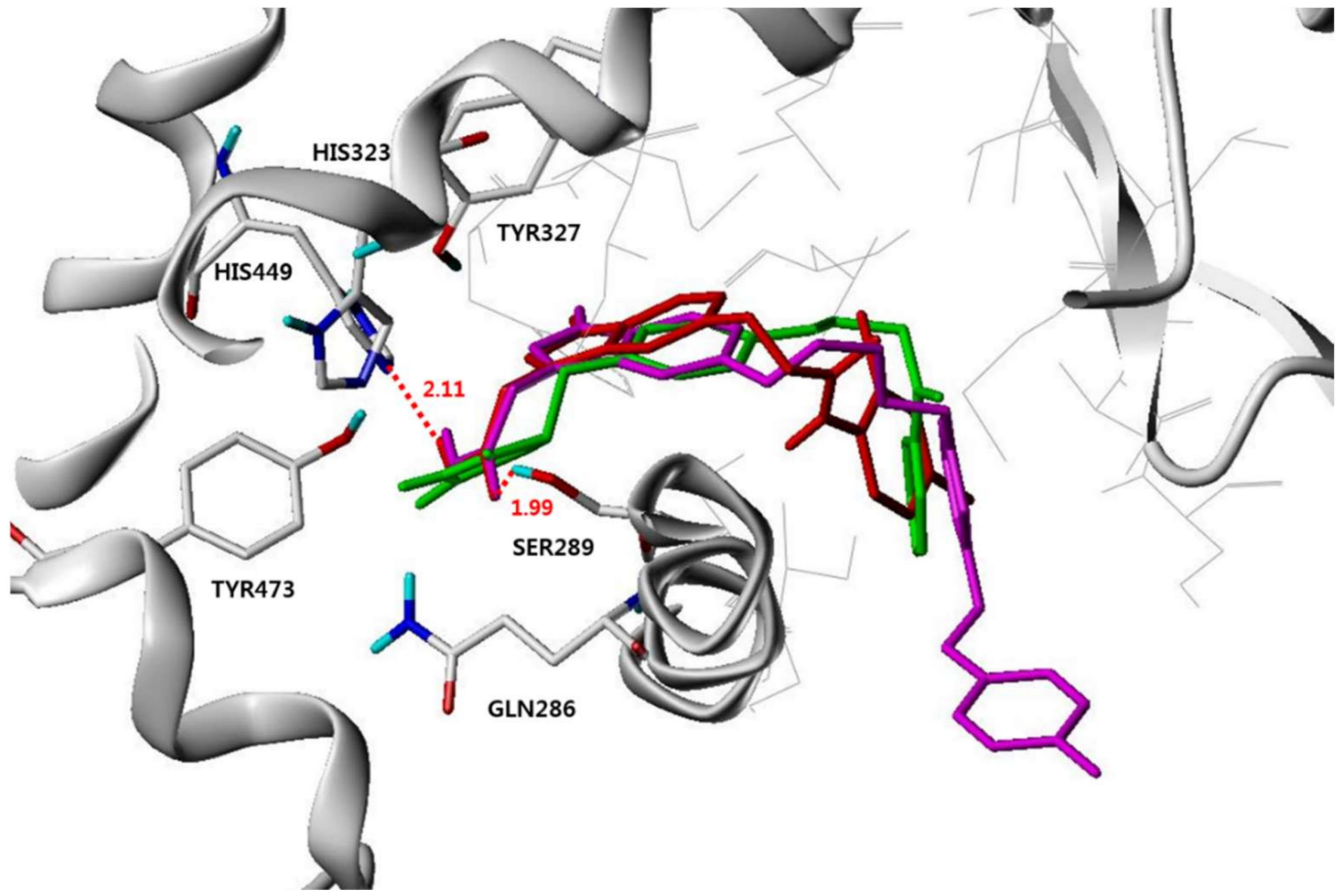
Binding modes of hit compound **1** (magenta) and compound **5** (red) in the active site of PPAR-γ LBD (PDB code: 2PRG; co-crystallized with rosiglitazone (green)). Only the critical amino acids that interacted with the docked ligands are displayed and labeled. Several residues for the key interaction (capped stick), hydrophobic residues (gray line), and hydrogen bonds (red-dotted lines) are depicted. The numbers indicate the atomic distance (Å).

To better understand the structural features of the synthesized compounds in the active site of the PPAR LBD, a docking study of compounds **1** and **5** was carried out using Surflex Dock interfaced with Sybyl-X (Figure 3). The crystal structure of PPAR-γ LBD complexed with the PPAR-γ selective agonist, rosiglitazone (PDB code: 2PRG) was selected to perform the docking of compounds [34]. The polar head groups of rosiglitazone (green) and compound **1** (magenta) revealed the H-bonding interaction with the polar residues in the hydrophilic pocket. The indole ring acted as an aromatic linker, similar to the benzene ring of rosiglitazone. Thus, the aromatic linker of each compound was well-overlapped in the docking position and made the compound U-shaped. The pyridine ring of rosiglitazone was located in the hydrophobic pocket of PPAR-γ and interacted with the hydrophobic amino acid residues. The benzene ring of compound **1** was well-aligned with the pyridine ring of rosiglitazone. The docking pose of compound **5** (red) might explain its low activity. The acidic head group could form hydrogen bonds, similar to those of compound **1**. The newly introduced 2,4-dichloroanilide moiety was located near the hydrophobic pocket; however, it was misaligned with the pyridine ring of rosiglitazone. Moreover, the benzene ring, a part of the indole moiety, did not superimpose with that of other compounds. This might due to the non-flexibility of the linker. The rigid carbonyl plane was located at the corner; thus, the linker could not turn smoothly. Structural modification was needed to compensate this rigidity. The *N*-benzyl group of compounds **7** and **8** might make additional interaction with the side pocket. Nevertheless, we tried to use this benzyl group as an aromatic linker to hold the polar acidic head.

*N*-Benzylated indole derivatives, which had a carboxylic acid moiety at the *ortho*-, *meta*-, or *para*-position of the benzyl group, were synthesized. Among the 5-alkoxy compounds, the *ortho*-carboxybenzyl-substituted compound **12** was the most active, whereas the *meta*-carboxybenzyl-substituted compound **15** showed the highest activity among the 6-alkoxy series. We supposed that the activity was also affected by the distance between the alkoxy and carboxyl groups. 5-Alkoxy was a little far from the benzyl-substituted amine in the indole structure (*pseudo-para*), and the *ortho*-positioned carboxyl group was the best to fit into the hydrophilic pocket. However, 6-alkoxy was at the *pseudo-meta* position of the indole structure, thus the carboxyl group at the *meta* position showed better activity. Molecular modeling guided us to introduce additional substituents at the 3-position of the indole moiety, which could be placed in the extra back pocket near the indole structure. Thus, 3-benzyl- or prenyl-substituted *N*-(2-carboxybenzyl)indole compounds were also prepared. Unexpectedly, all analogs (**18**–**21**) with a substitution at the 3-position of the indole moiety exhibited weak PPAR agonistic activity. 

**Figure 4:**
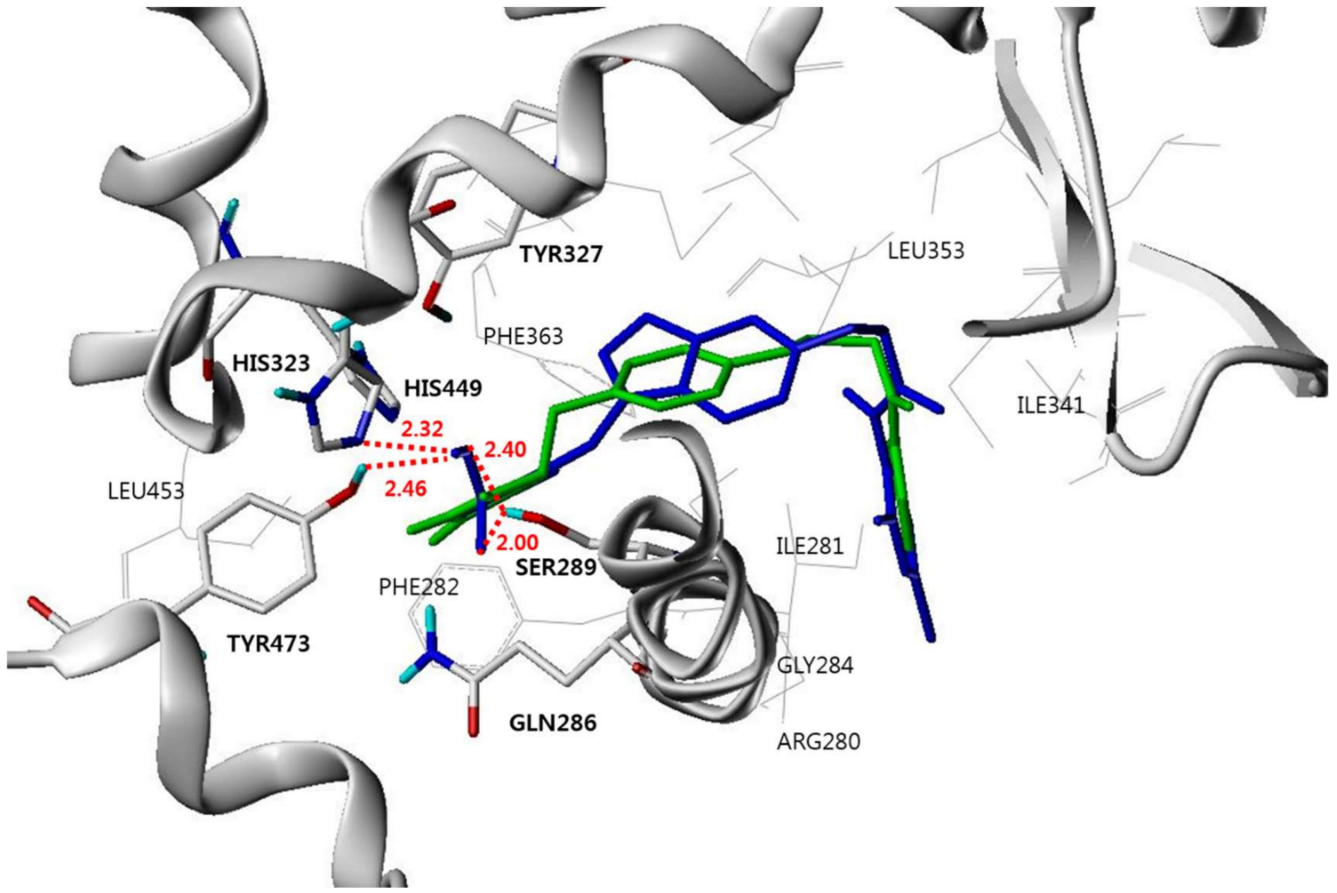
Binding modes of compound **12** (blue) in the active site of PPAR-γ LBD (PDB code: 2PRG; co-crystallized with rosiglitazone (green)). Only the critical amino acids that interacted with the docked ligands are displayed and labeled. Several residues for the key interaction (capped stick), hydrophobic residues (gray line), and hydrogen bonds (red-dotted lines) are depicted. The numbers indicate the atomic distance (Å).

A docking study of compound **12** was also carried out using the same system as described above. Compound **12** showed a U-shape binding mode wrapping around H3, similar to that of rosiglitazone (Figure 4). The carboxyl group of compound **12** was located in the hydrophilic pocket and interacted with the key polar residues, Ser289, His323, and Tyr473 by H-bonding. Next to the acidic head group, the benzene ring was positioned in the hydrophobic region formed by Phe282, Phe363, and Leu453. The indole ring acted as an aromatic linker, similar to the benzene ring of rosiglitazone. In addition, the dichloroanilide group was well-fitted in the hydrophobic pocket along with rosiglitazone. The indole group was located close to H3, and dichloroanilide was positioned between H3 and β2. Overall, the dichloroanilide moiety of compound **12** was well-aligned with the hydrophobic tail of rosiglitazone (Figure 4), in contrast to that of compounds **5** (Figure 3). The dichlorophenyl group of compound **12** could interact with the Ile281, Gly284, Ile341, and Leu353 residues, which form the hydrophobic pocket. In particular, the *ortho*-chloro atom was located 3.43 Å away from the carbonyl oxygen of Gly284 to make a halogen bonding interaction. Therefore, the high activity of compound **12** might be attributed to the proper binding and suitable interactions between compound **12** and PPAR-γ. 

**Figure 5:**
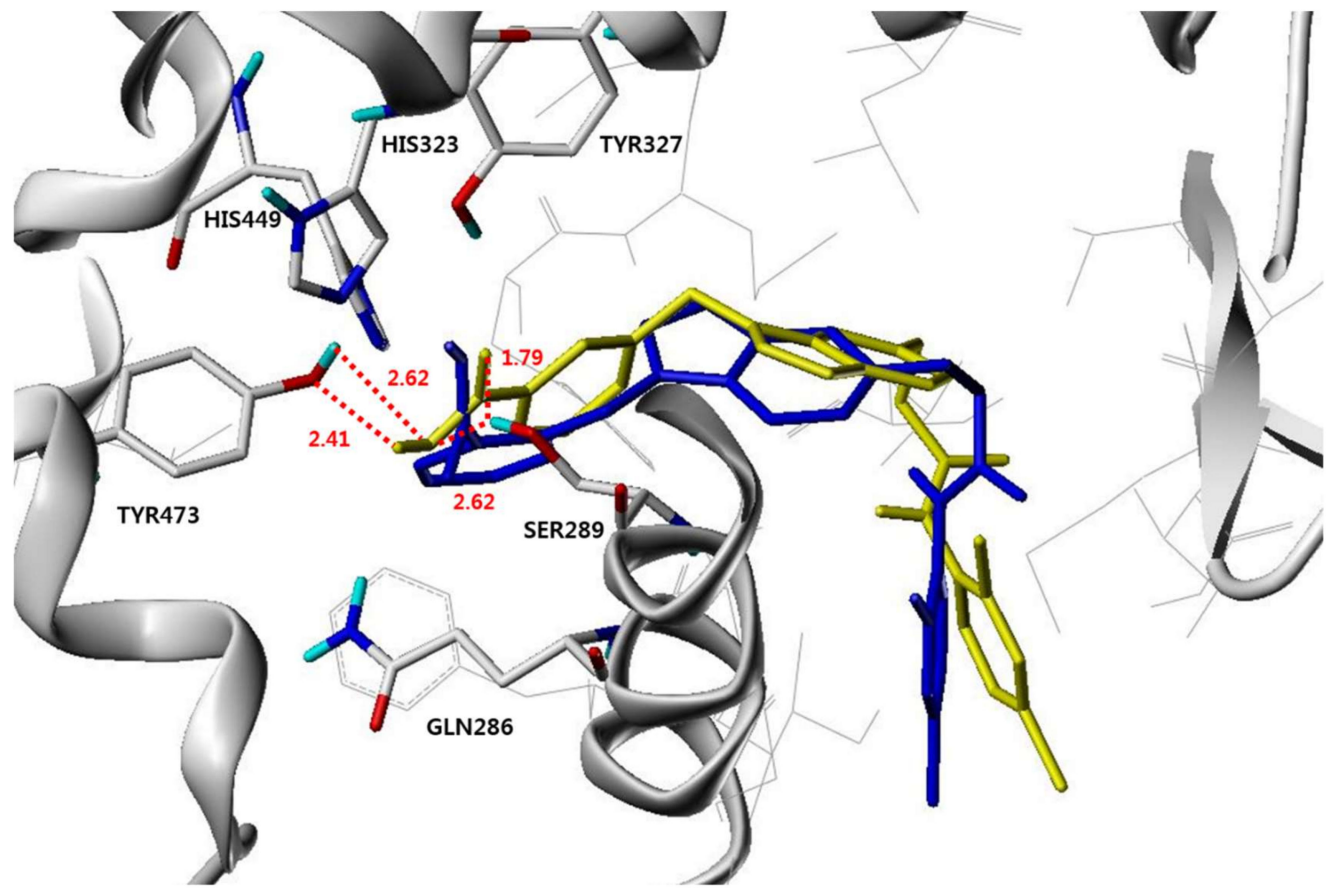
Binding modes of compound **12** (blue) and compound **15** (yellow) in the active site of PPAR-γ LBD (PDB code: 2PRG; co-crystallized with rosiglitazone). Only the critical amino acids that interacted with the docked ligands are displayed and labeled. Several residues for the key interaction (capped stick), hydrophobic residues (gray line), and hydrogen bonds (red-dotted lines) are depicted. The numbers indicate the atomic distance (Å).

Another potent compound, **15** belonged to the 6-alkoxyindole series. The carboxylic acid at the *meta* position of the *N*-benzyl group made 4 pairs of hydrogen bonds with the Ser289 and Tyr473 residues in the hydrophilic pocket (Figure 5). The carboxylic acid group of compound **15** was aligned similarly to that of compound **12**; however, the other part of compound **15** (from the benzyl group to the 2,4-dichloroanilide tail) was slightly moved back to locate the acid in the center of the hydrophilic region. Because of this movement, compound **15** was possibly less coordinated with the surrounding hydrophobic residues than compound **12**. The 6-alkoxyindole analog with *ortho*-carboxybenzyl group (13) and 5-alkoxyindole analog with *meta*-carboxybenzyl group (**14**) made less H-bonds with the polar residues because their position in 3D was influenced by the conformation of the whole molecule (data not shown). The findings of the docking study could explain the results of the in vitro transactivation assay. The substitution position of carboxylic acid on the benzyl group highly affected the PPAR activity, implying that the optimal positioning and matched combinations of the acidic head and lipophilic tail groups were essential. Taken together, we identified the *N*-(carboxylbenzyl)-substituted indole as a novel and promising scaffold for development of therapeutic agents targeting PPARs.

PPAR coactivator recruit assay was performed to determine the potency of the 4 most active compounds. Binding of agonist to the PPAR LBD causes a conformational change around helix 12 in the LBD, resulting in higher affinity for the coactivator peptide. When the terbium label on the anti-GST antibody complexed with PPAR is excited at 340 nm, energy is transferred to the fluorescein label on the coactivator peptide and detected as emission at 520 nm. The time-resolved fluorescence resonance energy transfer (TR-FRET) ratio of 520:495 is calculated and used to determine the EC_50_ from a dose response curve of the compound. Based on the biology of the PPAR-coactivator peptide interaction, this ligand EC_50_ is a composite value representing the amount of ligand required to bind to receptor, effect a conformational change, and recruit coactivator peptide. Potency of compounds show similar order to their activity (Table 3). Notably, compound **12** showed EC_50_ of 1.96 nM to PPAR-γ which was 1,970 fold selective to PPAR-α and 16,600 fold to PPAR-δ. The second potent compound **15** revealed EC_50_ of 32.3 nM to PPAR-γ whereas >100 μM to other PPAR subtypes. These results were huge improvement from our previous hit compounds (**1** and **2**), which exhibited PPAR-γ/δ dual agonistic activity at micromolar concentration. The PPAR-δ ligand binding pocket is significantly smaller than those of PPAR-α and PPAR-γ because of the narrowing of the pocket adjacent to the AF2 domain. Ligands such as TZDs show little or no binding to PPAR-δ. Compound **11** and **15** have *ortho* or *meta* substituted benzoic acid as an acidic head group. Their acidic head groups might seem to be too large to fit within the narrow PPAR-δ pocket. In contrast, the compound **1** and **2** contains an acetic acid head group that complements the narrow PPAR-δ ligand binding. And docking study of compound **11** with crystal structure of PPAR-α (PDB code: 3G8I; co-crystallized with aleglitazar) and PPAR-δ (PDB code: 3TKM; co-crystallized with GW0742) revealed misaligned conformation compared to their co-crystallized ligands, aleglitazar (PPAR-α/γ agonist) and GW0742 (PPAR-δ agonist) respectively (data not shown). The potency and selectivity of compound **11** could be explained by harmonization of steric effect of acidic head group, length and rigidity of hydrophobic tail, and properly tuned aromatic linker system.

## 3. Materials and Methods 

### 3.1. General Information

Most reagents and solvents were purchased from commercial sources and used without purification, with the following exceptions. Tetrahydrofuran (THF) was distilled from sodium benzophenone ketyl. Acetonitrile (MeCN), methylene chloride (CH_2_Cl_2_), dimethylformamide (DMF), and triethylamine were distilled from calcium hydride under nitrogen atmosphere. Column chromatography was performed using silica gel 60 (230–400 mesh, Merck) with the indicated solvents. Thin-layer chromatography (TLC) was performed using Kieselgel 60 F254 plates (Merck). ^1^H and ^13^C NMR spectra were recorded using a Varian Inova 400 or Bruker Avance III HD500 spectrometer using CDCl_3_, CD_3_OD, or (CD_3_)_2_SO as solvents. Chemical shifts (δ) were expressed in parts per million (ppm) downfield from an internal standard, tetramethylsilane. HRMS data were recorded using an Agilent 6530 QTOF mass spectrometer with an electrospray (ESI) interface with Agilent jet stream technology in the negative ion mode.

### 3.2. Experimental Procedures and Analytical Data of Compounds

#### 3.2.1. 2-Chloro-*N*-(2,4-Dichlorophenyl)Acetamide (3)

Triethylamine (1.1 equiv.) was added to a solution of 2,4-chloroaniline in CH_2_Cl_2_ (0.5 M) and stirred at room temperature for 30 min. Then, the reaction mixture was cooled to 0 °C. Chloroacetyl chloride (1.2 equiv.) was added dropwise while the temperature was maintained. After the completion of the reaction, water was added to the reaction mixture to quench the reaction. The organic layer was separated and washed with water thrice. Then, it was dried with brine and MgSO_4_, filtered, and concentrated under vacuum. The residue was purified by silica gel column chromatography using *n*-hexane and EtOAc (5:1) to yield a quantitative amount of the title compound **3**: ^1^H NMR (400 MHz, CDCl_3_) δ 8.89 (s, 1H), 8.34 (d, *J* = 8.8 Hz, 1H), 7.42 (d, *J* = 2.4 Hz, 1H), 7.27 (dd, *J* = 8.8, 2.4 Hz, 1H), 4.23 (s, 2H); ^13^C NMR (100 MHz, CDCl_3_) δ 164.0, 132.5, 130.2, 129.1, 128.1, 124.1, 122.0, 43.2.

#### 3.2.2. Synthetic Procedure for *O*-Alkylation of 5- or 6-Hydroxyindole Compounds

The prepared 5- or 6-hydoxyindole compounds were dissolved in anhydrous MeCN (0.5 M), and Cs_2_CO_3_ (1.5 equiv.) was added and stirred at room temperature for 30 min. Compound **3** (1.2 equiv.) was added to the reaction mixture then stirred until the reaction was complete. Then, the reaction mixture was diluted with water and extracted with EtOAc. The combined organic extracts were washed with water and brine, dried over anhydrous MgSO_4_, filtered, and concentrated under reduced pressure. The residue was purified by silica gel column chromatography using an appropriate mixture of *n*-hexane and EtOAc as an eluent to yield the corresponding alkoxyindole compounds (the methyl ester form of the final target compounds; yield = 65–86%).

**Methyl 2-(5-(2-((2,4-dichlorophenyl)amino)-2-oxoethoxy)-1*H*-indol-3-yl)acetate (P5):** ^1^H NMR (400 MHz, CDCl_3_) δ 9.12 (s, 1H), 8.46 (d, *J* = 8.9 Hz, 1H), 8.10 (s, 1H), 7.41 (d, *J* = 2.4 Hz, 1H), 7.31 (d, *J* = 8.8 Hz, 1H), 7.28 (dd, *J* = 8.8, 2.3 Hz, 1H), 7.20 (d, *J* = 2.2 Hz, 1H), 7.14 (d, *J* = 2.4 Hz, 1H), 6.96 (dd, *J* = 8.8, 2.4 Hz, 1H), 4.70 (s, 2H), 3.75 (d, *J* = 0.5 Hz, 2H), 3.70 (s, 3H); ^13^C NMR (100 MHz, CDCl_3_) δ 172.4, 167.2, 151.7, 132.9, 132.3, 129.6, 129.0, 128.1, 127.9, 124.6, 123.9, 122.1, 112.7, 112.5, 108.7, 103.1, 68.9, 52.2, 31.3.

**Methyl 2-(6-(2-((2,4-dichlorophenyl)amino)-2-oxoethoxy)-1*H*-indol-3-yl)acetate (P6):** ^1^H NMR (400 MHz, CDCl_3_) δ 9.06 (s, 1H), 8.42 (d, *J* = 8.9 Hz, 1H), 8.24 (s, 1H), 7.52 (d, *J* = 8.6 Hz, 1H), 7.38 (d, *J* = 2.3 Hz, 1H), 7.25 (dd, *J* = 8.7, 2.5 Hz, 1H), 7.07 (d, *J* = 2.2 Hz, 1H), 6.88 (dd, *J* = 8.6, 2.3 Hz, 1H), 6.84 (d, *J* = 2.0 Hz, 1H), 4.63 (s, 2H), 3.76 (s, 2H), 3.71 (s, 3H); ^13^C NMR (100 MHz, CDCl_3_) δ 172.7, 167.0, 153.8, 136.6, 132.7, 129.6, 129.0, 128.0, 123.8, 123.02, 122.97, 122.0, 120.1, 110.0, 108.5, 96.3, 68.4, 52.2, 31.2.

**Methyl 2-(1-benzyl-5-(2-((2,4-dichlorophenyl)amino)-2-oxoethoxy)-1*H*-indol-3-yl)acetate (P7):** ^1^H NMR (400 MHz, CDCl_3_) δ 9.11 (s, 1H), 8.46 (d, *J* = 8.9 Hz, 1H), 7.40 (d, *J* = 2.4 Hz, 1H), 7.32–7.24 (m, 4H), 7.18 (d, *J* = 8.9 Hz, 1H), 7.15 (d, *J* = 2.4 Hz, 1H), 7.14 (s, 1H), 7.12–7.09 (m, 2H), 6.92 (dd, *J* = 8.9, 2.5 Hz, 1H), 5.26 (s, 2H), 4.69 (s, 2H), 3.74 (d, *J* = 0.6 Hz, 2H), 3.70 (s, 3H); ^13^C NMR (100 MHz, CDCl_3_) δ 172.4, 167.2, 151.6, 137.3, 132.9, 132.8, 129.5, 128.98, 128.95, 128.6, 128.5, 128.1, 127.9, 126.9, 123.8, 122.1, 112.4, 111.2, 107.4, 103.4, 68.9, 52.2, 50.5, 31.2.

**Methyl 2-(1-benzyl-6-(2-((2,4-dichlorophenyl)amino)-2-oxoethoxy)-1*H*-indol-3-yl)acetate (P8):** ^1^H NMR (400 MHz, CDCl_3_) δ 9.00 (s, 1H), 8.41 (d, *J* = 8.9 Hz, 1H), 7.55 (d, *J* = 8.6 Hz, 1H), 7.38 (d, *J* = 2.4 Hz, 1H), 7.31–7.22 (m, 4H), 7.13–7.08 (m, 2H), 7.06 (s, 1H), 6.87 (dd, *J* = 8.6, 2.3 Hz, 1H), 6.76 (d, *J* = 2.2 Hz, 1H), 5.22 (s, 2H), 4.61 (s, 2H), 3.75 (s, 2H), 3.70 (s, 3H); ^13^C NMR (100 MHz, CDCl_3_) δ 172.5, 166.9, 153.8, 137.0, 132.8, 129.6, 129.0, 128.0, 127.9, 127.2, 126.9, 123.9, 123.8, 122.1, 120.5, 109.6, 107.8, 95.3, 68.5, 52.2, 50.3, 31.2.

**Methyl 2-((5-(2-((2,4-dichlorophenyl)amino)-2-oxoethoxy)-1*H*-indol-1-yl)methyl)benzoate (P12):** ^1^H NMR (400 MHz, CDCl_3_) δ 9.11 (s, 1H), 8.46 (d, *J* = 8.9 Hz, 1H), 8.12–7.98 (m, 1H), 7.39 (d, *J* = 2.3 Hz, 1H), 7.33–7.29 (m, 2H), 7.28–7.25 (m, 1H), 7.21 (d, *J* = 2.3 Hz, 1H), 7.17–7.12 (m, 2H), 6.90 (dd, *J* = 8.9, 2.4 Hz, 1H), 6.54 (d, *J* = 3.0 Hz, 1H), 6.48–6.44 (m, 1H), 5.76 (s, 2H), 4.68 (s, 2H), 3.94 (s, 3H); ^13^C NMR (100 MHz, CDCl_3_) δ 167.2, 151.6, 140.1, 133.1, 132.9, 132.7, 131.2, 130.1, 129.1, 129.0, 128.1, 127.8, 127.4, 127.1, 123.8, 122.1, 121.0, 112.2, 111.0, 104.9, 101.8, 95.9, 68.9, 52.4, 49.0.

**Methyl 2-((6-(2-((2,4-dichlorophenyl)amino)-2-oxoethoxy)-1*H*-indol-1-yl)methyl) benzoate (P13):** ^1^H NMR (400 MHz, CDCl_3_) δ 8.99 (s, 1H), 8.39 (d, *J* = 8.9 Hz, 1H), 8.07–8.02 (m, 1H), 7.59 (d, *J* = 8.6 Hz, 1H), 7.36 (d, *J* = 2.4 Hz, 1H), 7.30–7.26 (m, 2H), 7.25–7.22 (m, 1H), 7.08 (d, *J* = 3.2 Hz, 1H), 6.88 (dd, *J* = 8.6, 2.3 Hz, 1H), 6.73 (d, *J* = 2.2 Hz, 1H), 6.55 (dd, *J* = 3.2, 0.7 Hz, 1H), 6.49–6.44 (m, 1H), 5.73 (s, 2H), 4.59 (s, 2H), 3.95 (s, 3H); ^13^C NMR (100 MHz, CDCl_3_) δ 166.9, 153.6, 139.8, 136.9, 133.0, 132.8, 131.2, 129.5, 128.9, 128.7, 128.0, 127.8, 127.4, 127.0, 124.4, 123.8, 122.2, 122.0, 109.9, 102.0, 95.2, 68.5, 52.4, 48.8.

**Methyl 3-((5-(2-((2,4-dichlorophenyl)amino)-2-oxoethoxy)-1*H*-indol-1-yl)methyl) benzoate (P14):** ^1^H NMR (400 MHz, CDCl_3_) δ 9.10 (s, 1H), 8.45 (d, *J* = 8.9 Hz, 1H), 7.93 (d, *J* = 7.8 Hz, 1H), 7.89 (s, 1H), 7.38 (d, *J* = 2.3 Hz, 1H), 7.34 (t, *J* = 7.7 Hz, 1H), 7.25 (dd, *J* = 8.9, 2.3 Hz, 1H), 7.21–7.13 (m, 4H), 6.91 (dd, *J* = 8.8, 2.4 Hz, 1H), 6.51 (d, *J* = 2.6 Hz, 1H), 5.32 (s, 2H), 4.66 (s, 2H), 3.89 (s, 3H); ^13^C NMR (100 MHz, CDCl_3_) δ 167.0, 151.5, 137.8, 132.7, 132.3, 131.1, 130.7, 129.4, 129.3, 129.2, 128.99 128.95, 128.8, 127.92, 127.88, 123.6, 121.9, 112.0, 110.7, 104.9, 101.8, 68.7, 52.2, 50.1.

**Methyl 3-((6-(2-((2,4-dichlorophenyl)amino)-2-oxoethoxy)-1*H*-indol-1-yl)methyl) benzoate (P15):** ^1^H NMR (400 MHz, CDCl_3_) δ 8.98 (s, 1H), 8.39 (d, *J* = 8.9 Hz, 1H), 7.91 (d, *J* = 7.7 Hz, 1H), 7.88 (s, 1H), 7.57 (d, *J* = 8.6 Hz, 1H), 7.36 (d, *J* = 2.4 Hz, 1H), 7.32 (t, *J* = 7.7 Hz, 1H), 7.24 (dd, *J* = 8.9, 2.4 Hz, 1H), 7.18 (d, *J* = 7.7 Hz, 1H), 7.08 (d, *J* = 3.2 Hz, 1H), 6.86 (dd, *J* = 8.6, 2.3 Hz, 1H), 6.75 (d, *J* = 2.1 Hz, 1H), 6.52 (dd, *J* = 3.2, 0.8 Hz, 1H), 5.29 (s, 2H), 4.60 (s, 2H), 3.89 (s, 3H); ^13^C NMR (100 MHz, CDCl_3_) δ 166.8, 153.6, 137.6, 136.6, 132.7, 131.2, 130.9, 129.5, 129.10, 129.09, 128.9, 128.2, 128.03, 127.99, 124.5, 123.7, 122.3, 122.0, 109.8, 102.2, 95.1, 68.5, 52.3, 50.0.

**Methyl 4-((5-(2-((2,4-dichlorophenyl)amino)-2-oxoethoxy)-1*H*-indol-1-yl)methyl) benzoate (P16):** ^1^H NMR (400 MHz, CDCl_3_) δ 9.10 (s, 1H), 8.46 (d, *J* = 8.9 Hz, 1H), 7.96 (d, *J* = 8.2 Hz, 2H), 7.39 (d, *J* = 2.4 Hz, 1H), 7.27 (dd, *J* = 8.7, 2.1 Hz, 1H), 7.19 (d, *J* = 2.4 Hz, 1H), 7.16 (d, *J* = 3.1 Hz, 1H), 7.15–7.10 (m, 3H), 6.91 (dd, *J* = 8.9, 2.4 Hz, 1H), 6.53 (d, *J* = 3.1 Hz, 1H), 5.36 (s, 2H), 4.68 (s, 2H), 3.89 (s, 3H); ^13^C NMR (100 MHz, CDCl_3_) δ 167.2, 151.7, 142.6, 132.9, 132.5, 130.3, 129.8, 129.7, 129.5, 129.3, 129.0, 128.1, 126.6, 123.8, 122.1, 120.0, 112.3, 110.9, 105.1, 102.0, 68.8, 52.3, 50.3.

**Methyl 4-((6-(2-((2,4-dichlorophenyl)amino)-2-oxoethoxy)-1*H*-indol-1-yl)methyl) benzoate (P17):** ^1^H NMR (400 MHz, CDCl_3_) δ 8.97 (s, 1H), 8.38 (d, *J* = 8.9 Hz, 1H), 7.94 (d, *J* = 8.2 Hz, 2H), 7.58 (d, *J* = 8.6 Hz, 1H), 7.36 (d, *J* = 2.3 Hz, 1H), 7.24 (dd, *J* = 9.1, 2.1 Hz, 1H), 7.11 (d, *J* = 8.2 Hz, 2H), 7.09 (d, *J* = 3.2 Hz, 1H), 6.87 (dd, *J* = 8.6, 2.2 Hz, 1H), 6.72 (d, *J* = 1.9 Hz, 1H), 6.54 (d, *J* = 3.1 Hz, 1H), 5.32 (s, 2H), 4.60 (s, 2H), 3.89 (s, 3H); ^13^C NMR (100 MHz, CDCl_3_) δ 166.8, 153.7, 142.3, 136.7, 132.7, 130.3, 129.8, 129.6, 128.9, 128.3, 128.0, 126.6, 124.5, 123.7, 122.3, 122.0, 109.9, 102.3, 95.0, 68.5, 52.3, 50.1.

**Methyl 2-((3-benzyl-5-(2-((2,4-dichlorophenyl)amino)-2-oxoethoxy)-1*H*-indol-1-yl)methyl) benzoate (P18):** ^1^H NMR (400 MHz, CDCl_3_) δ 9.06 (s, 1H), 8.43 (d, *J* = 8.9 Hz, 1H), 8.07–7.99 (m, 1H), 7.38 (d, *J* = 2.4 Hz, 1H), 7.36–7.22 (m, 7H), 7.20–7.14 (m, 1H), 7.09 (d, *J* = 8.8 Hz, 1H), 7.02 (d, *J* = 2.3 Hz, 1H), 6.91–6.84 (m, 2H), 6.52–6.46 (m, 1H), 5.70 (s, 2H), 4.62 (s, 2H), 4.10 (s, 2H), 3.92 (s, 3H); ^13^C NMR (100 MHz, CDCl_3_) δ 167.5, 167.2, 151.3, 141.0, 140.3, 133.3, 133.0, 132.9, 131.1, 129.5, 129.0, 128.7, 128.52, 128.48, 128.42, 128.0, 127.8, 127.3, 127.0, 126.1, 123.8, 122.1, 114.9, 112.1, 111.0, 103.6, 68.9, 52.3, 48.8, 31.7.

**Methyl 2-((3-benzyl-6-(2-((2,4-dichlorophenyl)amino)-2-oxoethoxy)-1*H*-indol-1-yl)methyl) benzoate (P19):** ^1^H NMR (400 MHz, CDCl_3_) δ 8.98 (s, 1H), 8.38 (d, *J* = 8.9 Hz, 1H), 8.06–8.01 (m, 1H), 7.44 (d, *J* = 8.6 Hz, 1H), 7.37 (d, *J* = 2.3 Hz, 1H), 7.32–7.22 (m, 7H), 7.22–7.16 (m, 1H), 6.84–6.79 (m, 2H), 6.70 (d, *J* = 2.0 Hz, 1H), 6.54–6.49 (m, 1H), 5.67 (s, 2H), 4.58 (s, 2H), 4.11 (s, 2H), 3.94 (s, 3H); ^13^C NMR (100 MHz, CDCl_3_) δ 167.5, 166.9, 153.8, 141.2, 140.0, 137.5, 133.0, 132.8, 131.2, 129.6, 129.0, 128.8, 128.5, 128.0, 127.9, 127.4, 127.04, 126.95, 126.1, 124.0, 123.8, 122.1, 120.7, 115.4, 109.3, 95.3, 68.6, 52.4, 48.7, 31.7.

**Methyl 2-((5-(2-((2,4-dichlorophenyl)amino)-2-oxoethoxy)-3-(3-methylbut-2-en-1-yl)-1*H*-indol-1-yl)methyl) benzoate (P20):** ^1^H NMR (500 MHz, CDCl_3_) δ 9.01 (s, 1H), 8.40 (d, *J* = 8.9 Hz, 1H), 8.06–8.02 (m, 1H), 7.54 (d, *J* = 8.6 Hz, 1H), 7.38 (d, *J* = 2.3 Hz, 1H), 7.31–7.28 (m, 2H), 7.24 (d, *J* = 2.3 Hz, 1H), 6.86 (dd, *J* = 8.6, 2.2 Hz, 1H), 6.83 (s, 1H), 6.69 (d, *J* = 2.2 Hz, 1H), 6.52–6.48 (m, 1H), 5.67 (s, 2H), 5.45–5.40 (m, 1H), 4.60 (s, 2H), 3.96 (s, 3H), 3.45 (d, *J* = 6.8 Hz, 2H), 1.77 (s, 3H), 1.75 (s, 3H); ^13^C NMR (125 MHz, CDCl_3_) δ 167.4, 166.9, 153.7, 140.1, 137.4, 132.9, 132.7, 132.1, 131.1, 129.4, 128.9, 127.9, 127.7, 127.2, 126.9, 125.7, 123.9, 123.7, 122.9, 122.0, 120.4, 115.7, 108.9, 95.2, 68.5, 52.2, 48.5, 25.7, 24.1, 17.8.

**Methyl 2-((6-(2-((2,4-dichlorophenyl)amino)-2-oxoethoxy)-3-(3-methylbut-2-en-1-yl)-1*H*-indol-1-yl)methyl) benzoate (P21):** ^1^H NMR (500 MHz, CDCl_3_) δ 9.13 (s, 1H), 8.47 (d, *J* = 8.9 Hz, 1H), 8.07–8.01 (m, 1H), 7.41 (d, *J* = 2.3 Hz, 1H), 7.31 (dt, *J* = 6.1, 4.1 Hz, 2H), 7.28 (dd, *J* = 8.9, 2.3 Hz, 1H), 7.14 (d, *J* = 2.4 Hz, 1H), 7.09 (d, *J* = 8.8 Hz, 1H), 6.91 (s, 1H), 6.89 (dd, *J* = 8.8, 2.4 Hz, 1H), 6.50–6.46 (m, 1H), 5.70 (s, 2H), 5.43–5.38 (m, 1H), 4.70 (s, 2H), 3.95 (s, 3H), 3.44 (d, *J* = 7.0 Hz, 2H), 1.78 (s, 3H), 1.75 (s, 3H); ^13^C NMR (125 MHz, CDCl_3_) δ 167.5, 167.2, 151.1, 140.4, 133.2, 132.9, 132.8, 132.2, 131.0, 129.4, 128.9, 128.4, 127.9, 127.7, 127.3, 127.2, 127.0, 123.7, 122.8, 122.0, 115.2, 111.9, 110.8, 103.4, 68.9, 52.2, 48.6, 25.7, 24.1, 17.9.

#### 3.2.3. Synthetic Procedure for Hydrolysis of the Methyl Esters to Yield the Target Acid Compounds

LiOH**·**H_2_O (1.5 equiv.) was added to a solution of the alkoxyindole compounds in THF/MeOH/H_2_O (1 M, 2:1:1 *v*/*v*/*v*) and stirred at room temperature. After completing the reaction, the reaction mixture was concentrated under reduced pressure, acidified with 1N HCl solution, and extracted with EtOAc. The combined organic extracts were washed with water and brine, dried over anhydrous MgSO_4_, filtered, and concentrated under vacuum. The residue was purified by silica gel column chromatography using an appropriate mixture of CHCl_3_ and MeOH as an eluent to obtain the target compounds **6**–**9** and **13**–**22** with 59–92% yield.

**2-(5-(2-((2,4-Dichlorophenyl)amino)-2-oxoethoxy)-1*H*-indol-3-yl)acetic acid (5):** ^1^H NMR (400 MHz, CDCl_3_/CD_3_OD) δ 8.14 (d, *J* = 8.8 Hz, 1H), 7.54 (d, *J* = 2.2 Hz, 1H), 7.35 (dd, *J* = 8.8, 2.3 Hz, 1H), 7.31 (d, *J* = 8.8 Hz, 1H), 7.19 (s, 2H), 6.94 (dd, *J* = 8.6, 2.3 Hz, 1H), 4.73 (s, 2H), 3.71 (s, 2H); HRMS(ESI): m/z 391.0263 [M-H]^−^ (calcd for C_18_H_13_Cl_2_N_2_O_4_ = 391.0258).

**2-(6-(2-((2,4-Dichlorophenyl)amino)-2-oxoethoxy)-1*H*-indol-3-yl)acetic acid (6):** ^1^H NMR (400 MHz, CDCl_3_/CD_3_OD) δ 8.20 (d, *J* = 8.8 Hz, 1H), 7.75 (s, 1H), 7.50 (d, *J* = 8.7 Hz, 1H), 7.47 (d, *J* = 2.3 Hz, 1H), 7.31 (dd, *J* = 8.8, 2.4 Hz, 1H), 7.08 (s, 1H), 6.99 (d, *J* = 2.1 Hz, 1H), 6.83 (dd, *J* = 8.7, 2.3 Hz, 1H), 4.70 (s, 2H), 3.65 (s, 2H); HRMS(ESI): m/z 391.0256 [M-H]^−^ (calcd for C_18_H_13_Cl_2_N_2_O_4_ = 391.0258).

**2-(1-Benzyl-5-(2-((2,4-dichlorophenyl)amino)-2-oxoethoxy)-1*H*-indol-3-yl)acetic acid (7):** ^1^H NMR (400 MHz, CDCl_3_/CD_3_OD) δ 8.34 (d, *J* = 8.9 Hz, 1H), 7.50 (s, 1H), 7.45 (d, *J* = 2.3 Hz, 1H), 7.32 (d, *J* = 2.3 Hz, 1H), 7.31–7.26 (m, 2H), 7.24–7.18 (m, 3H), 7.13 (d, *J* = 6.6 Hz, 2H), 6.94 (dd, *J* = 9.2, 2.2 Hz, 1H), 5.30 (s, 2H), 4.72 (s, 2H), 3.74 (s, 2H); HRMS(ESI): m/z 481.0724 [M-H]^−^ (calcd for C_25_H_19_Cl_2_N_2_O_4_ = 481.0727).

**2-(1-Benzyl-6-(2-((2,4-dichlorophenyl)amino)-2-oxoethoxy)-1*H*-indol-3-yl)acetic acid (8):** ^1^H NMR (400 MHz, CDCl_3_/CD_3_OD) δ 8.25 (d, *J* = 8.8 Hz, 1H), 7.57 (d, *J* = 8.6 Hz, 1H), 7.44 (d, *J* = 2.2 Hz, 1H), 7.30 (dd, *J* = 8.8, 2.4 Hz, 1H), 7.28–7.21 (m, 3H), 7.16–7.11 (m, 3H), 6.89 (dd, *J* = 8.7, 2.0 Hz, 1H), 6.84 (d, *J* = 2.0 Hz, 1H), 5.29 (s, 2H), 4.66 (s, 2H), 3.74 (s, 2H); HRMS(ESI): m/z 481.0726 [M-H]^−^ (calcd for C_25_H_19_Cl_2_N_2_O_4_ = 481.0727).

**2-((5-(2-((2,4-Dichlorophenyl)amino)-2-oxoethoxy)-1*H*-indol-1-yl)methyl)benzoic acid (12):** ^1^H NMR (400 MHz, CDCl_3_/CD_3_OD) δ 8.38 (d, *J* = 8.9 Hz, 1H), 8.03–7.99 (m, 1H), 7.42–7.39 (m, 1H), 7.28 (dd, *J* = 8.9, 2.4 Hz, 1H), 7.24–7.20 (m, 2H), 7.19 (d, *J* = 2.4 Hz, 1H), 7.12 (d, *J* = 3.1 Hz, 1H), 7.10 (d, *J* = 8.9 Hz, 1H), 6.82 (dd, *J* = 8.8, 2.4 Hz, 1H), 6.49 (d, *J* = 2.6 Hz, 1H), 6.42–6.38 (m, 1H), 5.75 (s, 2H), 4.66 (s, 2H); HRMS(ESI): m/z 467.0549 [M-H]^−^ (calcd for C_24_H_17_Cl_2_N_2_O_4_ = 467.0571).

**2-((6-(2-((2,4-Dichlorophenyl)amino)-2-oxoethoxy)-1*H*-indol-1-yl)methyl)benzoic acid (13):** ^1^H NMR (400 MHz, CDCl_3_/CD_3_OD) δ 8.32 (d, *J* = 8.9 Hz, 1H), 8.09–8.05 (m, 1H), 7.60 (d, *J* = 8.6 Hz, 1H), 7.41 (d, *J* = 2.4 Hz, 1H), 7.39 (d, *J* = 0.7 Hz, 1H), 7.30–7.25 (m, 3H), 7.13 (d, *J* = 3.1 Hz, 1H), 6.89 (dd, *J* = 8.6, 2.2 Hz, 1H), 6.81 (d, *J* = 1.9 Hz, 1H), 6.55 (d, *J* = 3.2 Hz, 1H), 6.52–6.48 (m, 1H), 5.77 (s, 2H), 4.63 (s, 2H); HRMS(ESI): m/z 467.0550 [M-H]^−^ (calcd for C_24_H_17_Cl_2_N_2_O_4_ = 467.0571).

**3-((5-(2-((2,4-Dichlorophenyl)amino)-2-oxoethoxy)-1*H*-indol-1-yl)methyl)benzoic acid (14):** ^1^H NMR (400 MHz, CDCl_3_/CD_3_OD) δ 8.37 (d, *J* = 8.8 Hz, 1H), 7.99–7.90 (m, 3H), 7.44 (d, *J* = 2.3 Hz, 1H), 7.36 (t, *J* = 7.6 Hz, 1H), 7.30 (dd, *J* = 8.8, 2.4 Hz, 1H), 7.24–7.20 (m, 4H), 6.93 (dd, *J* = 8.9, 2.6 Hz, 1H), 6.51 (d, *J* = 3.2 Hz, 1H), 5.38 (s, 2H), 4.70 (s, 2H); HRMS(ESI): m/z 467.0558 [M-H]^−^ (calcd for C_24_H_17_Cl_2_N_2_O_4_ = 467.0571).

**3-((6-(2-((2,4-Dichlorophenyl)amino)-2-oxoethoxy)-1*H*-indol-1-yl)methyl)benzoic acid (15):** ^1^H NMR (400 MHz, CDCl_3_/CD_3_OD) δ 8.25 (d, *J* = 8.8 Hz, 1H), 7.94–7.87 (m, 2H), 7.55 (d, *J* = 8.6 Hz, 1H), 7.42–7.38 (m, 1H), 7.28 (s, 1H), 7.26 (dd, *J* = 8.8, 2.4 Hz, 1H), 7.20–7.15 (m, 1H), 7.10 (d, *J* = 2.7 Hz, 1H), 6.86 (dd, *J* = 8.6, 2.2 Hz, 1H), 6.82 (s, 1H), 6.48 (d, *J* = 3.1 Hz, 1H), 5.28 (s, 2H), 4.63 (s, 2H); HRMS(ESI): m/z 467.0557 [M-H]^−^ (calcd for C_24_H_17_Cl_2_N_2_O_4_ = 467.0571).

**4-((5-(2-((2,4-Dichlorophenyl)amino)-2-oxoethoxy)-1*H*-indol-1-yl)methyl)benzoic acid (16):** ^1^H NMR (400 MHz, CDCl_3_/CD_3_OD) δ 8.17 (d, *J* = 8.9 Hz, 1H), 7.89 (d, *J* = 8.4 Hz, 2H), 7.44 (d, *J* = 2.4 Hz, 1H), 7.28 (dd, *J* = 8.9, 2.3 Hz, 1H), 7.23 (d, *J* = 3.0 Hz, 1H), 7.20–7.16 (m, 2H), 7.11 (d, *J* = 8.1 Hz, 2H), 6.89 (dd, *J* = 8.9, 2.2 Hz, 1H), 6.45 (d, *J* = 3.2 Hz, 1H), 5.39 (s, 2H), 4.68 (s, 2H); HRMS(ESI): m/z 467.0545 [M-H]^−^ (calcd for C_24_H_17_Cl_2_N_2_O_4_ = 467.0571).

**4-((6-(2-((2,4-Dichlorophenyl)amino)-2-oxoethoxy)-1*H*-indol-1-yl)methyl)benzoic acid (17):** ^1^H NMR (400 MHz, CDCl_3_/CD_3_OD) δ 9.06 (s, 1H), 8.33 (dd, *J* = 8.9, 3.1 Hz, 1H), 7.96 (d, *J* = 8 Hz, 2H), 7.59 (d, *J* = 8.6 Hz, 1H), 7.41 (d, *J* = 2.3 Hz, 1H), 7.28 (dd, *J* = 8.8, 2.3 Hz, 1H), 7.16–7.11 (m, 3H), 6.89 (dd, *J* = 8.6, 2.2 Hz, 1H), 6.79 (d, *J* = 2.0 Hz, 1H), 6.54 (d, *J* = 3.2 Hz, 1H), 5.36 (s, 2H), 4.64 (s, 2H); HRMS(ESI): m/z 467.0549 [M-H]^−^ (calcd for C_24_H_17_Cl_2_N_2_O_4_ = 467.0571).

**2-((3-Benzyl-5-(2-((2,4-dichlorophenyl)amino)-2-oxoethoxy)-1*H*-indol-1-yl)methyl)benzoic acid (18):** ^1^H NMR (400 MHz, (CD_3_)_2_SO) δ 9.59 (s, 1H), 7.92 (d, *J* = 7.1 Hz, 1H), 7.88 (d, *J* = 9.1 Hz, 1H), 7.70 (s, 1H), 7.45 (d, *J* = 9.0 Hz, 1H), 7.35 (t, *J* = 7.6 Hz, 2H), 7.31–7.19 (m, 6H), 7.14 (d, *J* = 6.7 Hz, 1H), 7.10 (s, 1H), 6.85 (d, *J* = 9.2 Hz, 1H), 6.44 (d, *J* = 7.2 Hz, 1H), 5.72 (s, 2H), 4.71 (s, 2H), 4.03 (s, 2H); HRMS(ESI): m/z 557.1010 [M-H]^−^ (calcd for C_31_H_23_Cl_2_N_2_O_4_ = 557.1040).

**2-((3-Benzyl-6-(2-((2,4-dichlorophenyl)amino)-2-oxoethoxy)-1*H*-indol-1-yl)methyl)benzoic acid (19):** ^1^H NMR (400 MHz, (CD_3_)_2_SO) δ 10.43 (s, 1H), 7.80 (d, *J* = 8.7 Hz, 1H), 7.63 (d, *J* = 2.1 Hz, 1H), 7.56 (d, *J* = 6.7 Hz, 1H), 7.38 (dd, *J* = 8.7, 2.2 Hz, 1H), 7.30–7.20 (m, 6H), 7.17–7.10 (m, 2H), 7.09–7.00 (m, 2H), 6.70 (d, *J* = 6.5 Hz, 1H), 6.66 (dd, *J* = 8.5, 1.8 Hz, 1H), 5.66 (s, 2H), 4.74 (s, 2H), 3.99 (s, 2H); HRMS(ESI): m/z 557.1010 [M-H]^−^ (calcd for C_31_H_23_Cl_2_N_2_O_4_ = 557.1040).

**2-((5-(2-((2,4-Dichlorophenyl)amino)-2-oxoethoxy)-3-(3-methylbut-2-en-1-yl)-1*H*-indol-1-yl)methyl)benzoic acid (20):** ^1^H NMR (400 MHz, CDCl_3_/CD_3_OD) δ 8.33 (1H, d, *J* = 8.8 Hz), 8.09 (1H, dd, *J* =5.6, 3.6 Hz), 7.54 (1H, d, *J* = 8.8 Hz), 7.41 (1H, d, *J* = 2.0 Hz), 7.31–7.25 (3H, m), 6.88–6.85 (2H, m), 6.75 (1H, d, *J* = 2.0 Hz), 6.50 (1H, dd, *J* = 5.6, 3.2 Hz), 5.70 (2H, s), 5.43 (1H, t, *J* = 7.2 Hz), 4.62 (2H, s), 3.46 (2H, d, *J* = 6.8 Hz), 1.77 (3H, s), 1.76 (3H, s); HRMS(ESI): m/z 535.1178 [M-H]^−^ (calcd for C_29_H_25_Cl_2_N_2_O_4_ = 535.1197).

**2-((6-(2-((2,4-Dichlorophenyl)amino)-2-oxoethoxy)-3-(3-methylbut-2-en-1-yl)-1*H*-indol-1-yl)methyl)benzoic acid (21):** ^1^H NMR (400 MHz, CDCl_3_/CD_3_OD) δ 8.31 (1H, d, *J* = 8.8 Hz), 8.08–8.05 (1H, m), 7.55 (1H, d, *J* = 8.4 Hz), 7.42 (1H, d, *J* = 2.4 Hz), 7.31–7.26 (3H, m), 6.89–6.86 (2H, m), 6.78 (1H, d, *J* = 2.0 Hz), 6.52-6.49 (1H, m), 5.71 (2H, s), 5.44 (1H, t, *J* = 7.2 Hz), 4.64 (2H, s), 3.47 (2H, d, *J* = 7.2 Hz), 1.78 (3H, s), 1.77 (3H, s); HRMS(ESI): m/z 535.1177 [M-H]^−^ (calcd for C_29_H_25_Cl_2_N_2_O_4_ = 535.1197).

### 3.3. In Vitro PPAR Transactivation Assay

Transient transfection and luciferase assay was carried out. CV-1 cells were seeded in 48-well plates at a density of 1.5 × 10^5^ cells/well in Dulbecco’s modified Eagle’s medium (DMEM) with containing 10% fetal bovine serum (FBS). The cells were transiently transfected with plasmid mixtures containing PPAR-α/γ expression vector and tk-PPRE-luciferase (Luc) vector for 6 h and then treated with the samples for 24 h. HEK293t cells were seeded in a 60-mm dish at a density of 1.5 × 10^6^ cells/dish. After transfection with the plasmid mixtures containing PPAR-δ expression vector and Luc vector for 6 h, the cells were transferred to a 96-well plate and treated with the samples. To normalize the transfection efficiency, a β-galactosidase plasmid was cotransfected. The luciferase activity in the cell lysates was measured using a luciferase assay system (Promega Corp., Madison, WI, USA), and the β-galactosidase activity was determined by measuring the absorbance at 410 nm using an ELISA plate reader. The data were expressed as the relative luciferase activity divided by the β-galactosidase activity. All constructs were kindly gifted by Dr. Ronald M. Evans at The Salk Institute (La Jolla, CA, USA).

### 3.4. Molecular Modeling

All molecular modeling calculations were carried out using Surflex Dock interfaced with SYBYL-X software, version 2.1.1 running on a system with Window 7 Home Premium K 64-bit OS. In this automated docking program, the flexibility of the ligands, proteins, and biomolecules was considered. The ligand was built in an incremental fashion, where each new fragment was added in all possible positions and conformations to a pre-placed base fragment inside the active site. All the molecules used for docking were sketched in SYBYL, and the energy minimizations were performed using Tripos Force Field and Gasteiger–Huckel charge with 100,000 iterations of the conjugate gradient method with a convergence criterion of 0.05 kcal/mol. To prepare the proteins, all hydrogens and MMFF94 charges were added, and the side-chain amides were fixed. A staged minimization was performed using Tripos Force Field and MMFF94 charge with 10,000 iterations of the Powell method with a convergence criterion of 0.5 kcal/mol without the initial optimization. The 3D coordinates of the active sites were determined from the X-ray crystal structures of PPAR-γ (PDB code: 2PRG), reported as a complex with rosiglitazone.

### 3.5. In vitro PPAR Coactivator Recruit Assay

EC_50_ of compounds were determined by recruitment of transcriptional coactivators using the TR-FRET based Lanthascreen coactivator assays (Invitrogen, Carlsbad, CA, USA). Which was serviced by Thermo Fisher Scientific (Medicon, WI, USA) according to the LanthaScreen TR-FRET coregulator protocol and assay conditions. Coregulator peptide PGC1a, TRAP220/DRIP2 and C33 was used for the coactivator recruit assay of PPAR-α, -γ, and -δ, respectively.

## 4. Conclusions

In conclusion, we designed and synthesized several dichloroanilide-linked indole derivatives and examined their potent and selective PPAR activity. The PPAR transcriptional activities of these compounds were tested using a cell-based reporter assay. Compound **12** exhibited significant activity as a PPAR-γ agonist, with approximately 2-fold higher PPAR-γ activity than that of pioglitazone. PPAR coactivator recruit assay revealed EC_50_ of compound **12** with 1.96 nM. And it showed 1970-fold selectivity to PPAR-α and 16,600-fold to PPAR-δ. The docking study provided structural insights of compound **12** in association with PPAR-γ and rationalized its good PPAR-γ agonistic activity. Therefore, the *N*-(carboxybenzyl)indole was suggested as an original framework for the next generation of PPAR ligands. In addition, our scaffold tuning approach could be an expeditious way to optimize the activity of compounds. This novel PPAR-γ agonist and scaffold tuning approach is expected to broaden the bottleneck of new drug discovery for CVD, CML and various types of solid tumors as well as metabolic diseases.

## Figures and Tables

**Table 1:** In vitro cell-based PPAR transactivation activity ^a^.

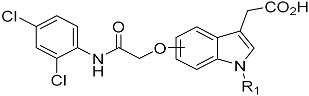					
Cpd. No.	Alkoxy position	R1	% max ^b^		
			PPAR-α	PPAR-γ	PPAR-δ
5	5	H	−38.13 ± 0.06	5.60 ± 0.14	11.84 ± 0.35
6	6	H	1.12 ± 0.24	8.66 ± 0.23	8.27 ± 0.33
7	5	benzyl	21.41 ± 0.21	31.20 ± 0.38	44.35 ± 0.77
8	6	benzyl	31.68 ± 0.22	71.87 ± 0.91	65.41 ± 0.70

^a^ The agonistic activity of the compounds (10 µM) was assayed on PPAR-Luc in CV-1 cells; ^b^ Relative activation with respect to the maximum activation obtained with GW7647 (10 µM), pioglitazone (10 µM), and GW0742 (10 μM) for PPAR-α, -γ, and -δ, respectively.

**Table 2:** In vitro cell-based PPAR transactivation activity ^a^.

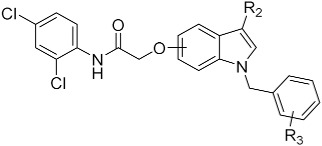						
Cpd. No.	Alkoxy position	R2	R3	% max ^b^		
				PPAR-α	PPAR-γ	PPAR-δ
12	5	H	2-CO_2_H	105.08 ± 0.41	189.21 ± 1.67	94.86 ± 0.20
13	6	H	2-CO_2_H	51.84 ± 0.25	74.54 ± 0.94	86.70 ± 0.53
14	5	H	3-CO_2_H	10.33 ± 0.07	42.01 ± 0.52	23.85 ± 0.51
15	6	H	3-CO_2_H	78.15 ± 0.04	126.55 ± 0.92	86.09 ± 0.31
16	5	H	4-CO_2_H	-19.98 ± 0.08	0.29 ± 0.06	-1.07 ± 0.14
17	6	H	4-CO_2_H	-14.44 ± 0.16	-0.84 ± 0.16	4.87 ± 0.24
18	5	benzyl	2-CO_2_H	23.79 ± 0.11	93.20 ± 0.59	75.88 ± 0.35
19	6	benzyl	2-CO_2_H	7.25 ± 0.12	19.03 ± 0.13	19.40 ± 0.54
20	5	prenyl	2-CO_2_H	-5.93 ± 0.14	5.10 ± 0.20	8.92 ± 0.48
21	6	prenyl	2-CO_2_H	-2.30 ± 0.19	9.55 ± 0.32	22.16 ± 0.59

^a^ The agonistic activity of the compounds (10 µM) was assayed on PPAR-Luc in CV-1 cells; ^b^ Relative activation with respect to the maximum activation obtained with GW7647 (10 µM), pioglitazone (10 µM), and GW0742 (10 μM) for PPAR-α, -γ, and -δ, respectively.

**Table 3:** In vitro PPAR coactivator recruit assay ^a^.

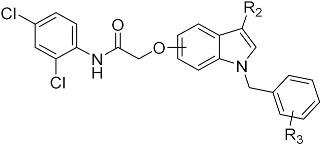						
Cpd. No.	Alkoxy position	R2	R3	EC_50_ (μM)		
				PPAR-α	PPAR-γ	PPAR-δ
12	5	H	2-CO_2_H	3.87	0.00196	32.6
13	6	H	2-CO_2_H	16.9	0.571	>100
15	6	H	3-CO_2_H	>100	0.0326	>100
18	5	benzyl	2-CO_2_H	>100	>11.1	28.1

^a^ The agonistic activity of the compounds was determined using TR-FRET coactivator recruit assay.
